# Interaction between somatostatin analogues and targeted therapies in neuroendocrine tumor cells

**DOI:** 10.1371/journal.pone.0218953

**Published:** 2019-06-25

**Authors:** Sebastian Krug, Jan-Philipp Mordhorst, Fabian Moser, Katharina Theuerkorn, Claudia Ruffert, Maren Egidi, Anja Rinke, Thomas M. Gress, Patrick Michl

**Affiliations:** 1 Department of Internal Medicine I, Martin Luther University Halle/Wittenberg, Halle (Saale), Germany; 2 Department of Gastroenterology and Endocrinology, Philipps-University, Marburg, Germany; Duke University School of Medicine, UNITED STATES

## Abstract

Somatostatin analogues (SSA) represent the standard of care for symptom control in patients with functional gastro-entero-pancreatic neuroendocrine tumors (GEP-NET). In addition, SSA exert significant anti-proliferative effects in mid-gut and pancreatic NET (PanNET). In parallel, molecularly targeted therapies (MTT) have been shown to improve progression free survival (PFS) in patients with PanNET. However, due to either primary or acquired resistance to MTT, their impact on overall survival (OS) remains unclear. To date, various hypotheses exist to explain differences in patient responsiveness to SSA and MTT. However, data addressing one of the most pivotal questions, whether combining SSA with novel MTT will result in synergistic or additive efficacy compared to monotherapy, are lacking. The aim of this study is to characterize the interaction, optimal sequence and dosing of SSA-based and molecularly targeted therapies in PanNET. Somatostatin receptor subtypes 1–5 (SSTR) were evaluated in the neuroendocrine cell lines Bon1, QGP1 and Ins-1 via immunoblot and qRT-PCR. The impact of the SSA-analogue lanreotide alone or in combination with the MTT sunitinib, everolimus and regorafenib on intracellular signalling, hormone secretion and cell proliferation was determined in cell lysates and supernatants. In addition, synergistic effects of SSA and MTT in various sequential therapeutic approaches were investigated. SSTR were differently expressed in the examined neuroendocrine tumor cell lines. SSTR modulation via lanreotide moderately influenced proliferation, mainly via modulating AKT and ERK signalling, which was paralleled by decreased chromogranin A (CgA) expression and secretion. Interestingly, MTT treatment with regorafenib upregulated the expression of SSTR-2 and -5, while sunitinib and everolimus did not significantly alter SSTR expression. Cell viability was significantly reduced by all MTT, with regorafenib exerting the most significant effects. However, compared to the marked effects of MTT alone, synergistic effects of combined MTT and lanreotide treatment were only modest and time- and dose-dependent. SSTR are differentially expressed in various NEN cell lines. Their expression is influenced by MTT treatment. Various sequential or simultaneous combinations of lanreotide and MTT did not lead to significant synergistic effects.

## Introduction

Neuroendocrine tumors of the gastro-entero-pancreatic system (GEP-NET) represent a rare and heterogeneous disease [1, 2]. While symptoms frequently occur late, the majority of NET patients are diagnosed with metastatic disease [3, 4]. Surgery remains the standard treatment for localized stages. In case of unresectable and metastatic disease medical treatment has shown to improve the long-term outcome of patients [5].

GEP-NET are characterized by the expression of somatostatin receptors (SSTR). Five SSTR subtypes have been described (SSTR 1–5), among them the SSTR2 (SSTR2A and SSTR2B) and 5 (SSTR5TDM4 and SSTR5TDM5) with different splice variants [6]. The SSTR2A is exclusively expressed in human tissue and a membrane-bound receptor, whereas SSTR1, 3 and 5 are located intracellularly [7]. SSTR are able to heterodimerize with other members of the SSTR family as well as with unrelated G-protein-coupled receptors and mediate several indirect and direct tumor effects such as cell cycle induction, apoptosis, modulation of angiogenesis and the immune system and controlling growth factor and hormone release [8]. In GEP-NET SSTR2 and 5 are preferentially implicated in diagnostic and therapeutic approaches. Previous data indicated a correlation of SSTR2 expression, differentiation and prognosis in GEP-NET patients [9, 10].

In this context, somatostatin analogues (SSA) are well established anti-secretory drugs that have been used as first line treatment for symptomatic control in hormonally active neuroendocrine tumors (NET) for three decades [11]. In addition to their pivotal role in symptom control, somatostatin analogues also demonstrated growth-inhibitory effects *in vitro* and *in vivo*. The anti-proliferative activities of SSA are mediated via two general signaling pathways: the inhibition of growth factor induced MAPK phosphorylation (blocking of the PI3K/Akt and GSK3ß pathway) and the activation of protein tyrosine phosphatases, such as the Src homology phosphatases, SHP-1 and SHP-2 [6]. To date, the SSTR2 is the best studied mediator of this mechanism. However, SSTR5 can suppress tumor cell proliferation by influencing the src-like tyrosine kinase p60src and phospholipase C [6].

A significant anti-tumor activity of SSAs was documented in two prospective randomized placebo-controlled trials in recent years. The PROMID study [12] compared octreotide LAR 30mg every 4 weeks versus placebo in 85 treatment-naïve patients with well-differentiated midgut NETs. The primary endpoint, time to tumour progression (TTP), in the octreotide LAR group was significantly longer compared to the placebo group (14.3 months vs. 6 months; HR 0.34; p = 0.000072). The most favourable results were seen in patients with low hepatic tumour load (<10% liver involvement).

More recently, the phase III CLARINET trial expanded the role of SSAs for tumor control in NET [13]: Patients with well- or moderately differentiated, non-functioning, somatostatin receptor-positive GEP-NETs with a Ki-67 of <10% were randomized to receive either lanreotide autogel 120 mg every 4 weeks or placebo. The vast majority of patients (96%) had stable disease at randomization. Lanreotide was associated with a significant prolongation of PFS with estimated PFS rates of 65.1% in the lanreotide group at 24 months versus 33% in the placebo group (HR 0.47; p<0.001). The benefit in patients with midgut NET (HR 0.35; p = 0.009) was greater than in the pancreatic subset (HR 0.58; p = 0.06). However, it is important to note that the study was not powered for statistical significance of the subgroups.

Besides SSA, a plethora of therapeutic options are available in advanced NET patients. Loco-ablative and loco-regional approaches may affect a predominantly localized liver burden. Further systemic options include targeted therapies such as sunitinib and everolimus or the peptide receptor radionuclide therapy (PRRT) [14–16]. It is an ongoing debate how to select patients for the best treatment and sequence. To date, no comparative prospective trials are available which address the optimum therapy sequence. In addition, no mechanistic studies are available investigating the molecular interactions between SSA-based therapies and MTT in a comprehensive manner. In this respect, clinical studies pointed out a PFS benefit, when the MTT everolimus was added to SSA. Two studies observed encouraging mPFS rates ranging between 16–33 months for the combination group, in comparison to the well-known everolimus monotherapy effect of approximately 11 months [17, 18].

The aim of this study is to characterize the interactions and optimal sequence of SSA-based therapies and MTT in appropriate preclinical in-vitro models of pancreatic neuroendocrine tumor cell lines. We hypothesized that MTT modulate the SSTR expression and thus affect the impact of SSA.

## Material and methods

### Material and cell lines

All media contained 10% fetal bovine serum and 40 μg/ml gentamicin. The following cell lines were cultured in the indicated media: human Bon1 carcinoid cells, a kind gift of R. Göke, University of Marburg, Germany and human QGP1 NET cells: DMEM/HAM’s F12 medium; rat Ins-1 insulinoma cells, donated by C. B. Wollheim, University of Geneva, Switzerland: RPMI1640 medium supplemented with additional 1 mM sodium pyruvate, 10 mM HEPES and 0.05 mM 2-ß-mercaptoethanol. All cells were cultured in a humidified atmosphere containing 5% CO2 at 37°C. Cell number was analyzed by counting the cells in a Neubauer chamber. Lanreotide was provided by Ipsen Pharma, everolimus and sunitinib were purchased from Santa Cruz Biotechnologies. Regorafenib was provided by Bayer.

### RNA isolation, cDNA synthesis, and real-time PCR

RNA was extracted using the RNeasy Mini Kit (Qiagen), and first-strand cDNA was synthesized using random hexamer primers and Superscript II reverse transcriptase (Invitrogen). Quantitative RT-PCR analysis was performed with an Applied Biosystems 7500 Fast Real time PCR using the SYBR Green PCR Master Mix kit (Applied Biosystems, Wellesley, Massachusetts, USA) according to the manufacturer’s instructions. Sequence specific primer pairs were designed using the Primer Express software (Applied Biosystems). RPLP0 was used as internal standard (forward 5’-GTC GGA GGA GTC GGA CGA G-3’; reverse 5’-GCC TTT ATT TCC TTG TTT TGC AAA-3’). Primer sequences for SSTR1 are forward 5'-ttt ccc tac cct gca act tc-3' and reverse 5'-cgt ggc tca gct taa aca aa-3'; for SSTR2 are forward 5'-caa cca aca cct caa acc ag-3' and reverse 5'-gca tag cgg agg atg aca ta-3'; SSTR3 are forward 5'-gac ccc cgg tga aat cct t-3' and reverse 5'-agg tgt gct gaa gat cca agt ct-3'; SSTR4 are forward 5'-acg tgt ccc tta tcc tta gct atg c-3' and reverse 5'-cag aga acc cgc tgg aag aa-3'; SSTR5 are forward 5'-ttc gtg gtc atc ctc tcc ta-3' and reverse 5'-gag agg aag ccg tag agg ac-3'.

### Immunoblotting

Cells were lysed in RIPA buffer supplemented with complete protease inhibitor cocktail (Roche, Mannheim, Germany). Nuclear and cytoplasmic fractions were performed using the ProteoJET Cytoplasmic and Nuclear Protein Extraction Kit (Fermentas, St. Leon-Rot, Germany). Immunoblots were performed as described previously [19]. Supernatant was harvested 24h and 48h after treatment. Immunoblots were probed with primary antibodies against SSTR1-5 (abcam, Cambridge, UK), PARP, AKT, pAKT, ERK, pERK, pRPS6, RPS6, eiF4E, PCNA, Cyclin D1, CDK4, chromogranin A and beta-actin (Sigma-Aldrich). Peroxidase-conjugated secondary antibodies against mouse or rabbit were obtained from Amersham (Freiburg, Germany) and goat from Sigma. Blots were detected by Western Lightning ECL (PerkinElmer, Rodgau, Germany).

### Viability, proliferation and apoptosis assays

Cell viability was determined either by using the MTT Cell Proliferation Kit (Roche Diagnostics, Mannheim, Germany) or by using CellTiter-Glo Luminescent Cell Viability Assay (Promega) according to the manufacturer’s instructions. Whereas lanreotide was diluted in H20, everolimus, sunitinib and regorafenib were diluted in DMSO. DMSO and mock (H20) served as treatment control for the targeted therapies and lanreotide, respectively. For MTT assays, 30.000 cells per well (12-well plate) were used and treatment comprised the given drugs for a period of 24h, 48h or 72h. Treatment was started 24h after seeding. For ATP assays, 7.500 cells per well (96-well plate) were used and treatment was initiated as described above. Protein lysate and mRNA collection was performed from cells treated in 6-well plates. For this purpose 300.000 cells per well were seeded. Treatment protocols were as follows: lanreotide 10nM, 100nM, 1μM and 10μM; regorafenib 2μM, 4μM, 6μM, 8μM and 10μM; everolimus 1nM, 10nM, 100nM and 1000nM; sunitinib 500nM, 1μM, 2,5μM and 5μM. In proliferation assays, mock and DMSO was used as internal control for the targeted therapies to rule out significant DMSO-dependent effects.

### Statistical design and analysis

All data are presented as mean ± standard deviation. Two-tailed paired Student’s t test was used for statistical evaluation of the data. A p value < 0.05 was considered significant. All statistical calculations were performed using SPSS (IBM SPSS Statistics) and GraphPad Prism.

## Results

### SSTR expression in different NET cell lines

The human pancreatic NET cell lines Bon1, QGP1 and the rat insulinoma cell line Ins-1 were used to detect differences in SSTR expression patterns on RNA and protein level (S1A and S1B Fig). In Bon1 cells SSTR1 and 5 were strongly expressed, whereas SSTR2-4 showed a low basal expression. A similar pattern was seen in QGP1 cells which, however, exhibited a lower protein expression of SSTR2 and -5 compared to Bon1 cells. In contrast, Ins-1 cells exhibit a strong SSTR2 expression on mRNA and protein level. Given their human origin Bon1 and QGP1 cells were used for further experiments. Based on the low basal expression of SSTR3 and -4 in all cell lines, we focused on SSTR1,-2 and -5 in our further experiments.

### Influence of lanreotide and MTT on SSTR expression in Bon1 and QPG1 cells

**Fig 1:**
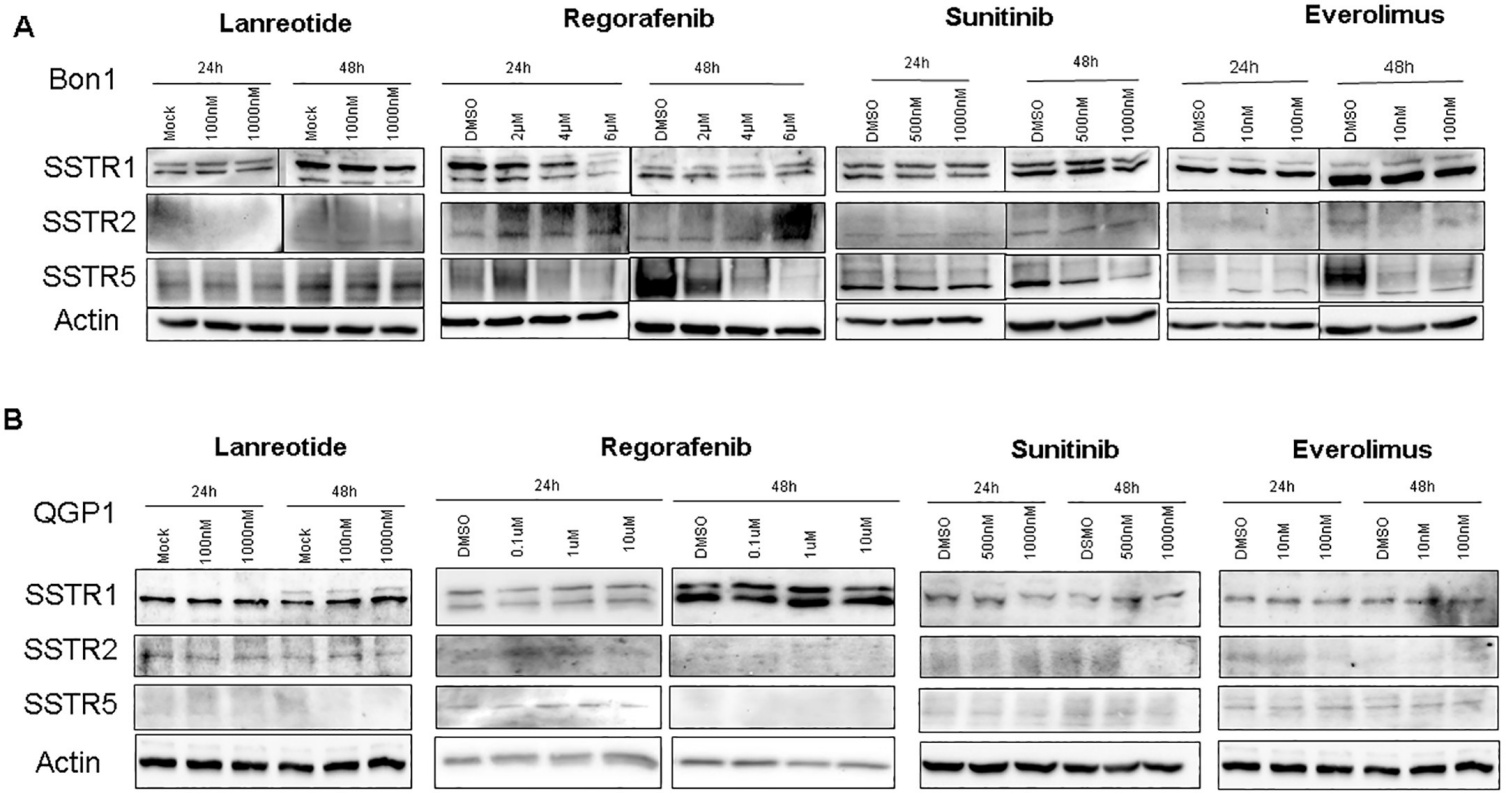
Representative western blots of SSTR1, 2 and 5 in Bon1 and QGP1 cells treated with lanreotide, regorafenib, everolimus and sunitinib in different doses. Protein lysates were collected after 24h and 48h. Data are representative for at least three independent experiments. ß-actin served as internal control.

Treatment with lanreotide did not lead to alterations in the expression of SSTR subtypes 1, 2 and 5 on protein level (Fig 1) and mRNA level (S2 and S3 Figs) in Bon1 (Fig 1A) and QGP1 cells (Fig 1B). In contrast, MTT’s induced distinct alterations in SSTR expression patterns in Bon1 cells (Fig 1A): Regorafenib transiently induced SSTR2 and, in low doses, also SSTR5 expression after 24h treatment, followed by a subsequent decrease of SSTR5 after 48h, while SSTR2 remained upregulated. In contrast, SSTR1 was consistently reduced at both time points after addition of regorafenib. Sunitinib and everolimus similarly led to a reduction of SSTR5, whereas SSTR1 and -2 showed no consistent regulation (Fig 1A). Interestingly, on mRNA level the SSTR regulation by sunitinib and everolimus revealed similar trends. However, the upregulation of SSTR2 and -5 by regorafenib was not seen on mRNA level which may indicate that the observed transient regulations might be due to post-transcriptional modifications, altered protein stability or a secondary effect due to reduced cell viability. In contrast to the findings in Bon1 cells, there was no consistent SSTR regulation detectable in QGP1 cells (Fig 1B and S3 Fig) indicating that SSTR regulation by MTT is cell type-specific and no long-term regulations of SSTR expression patterns are induced by MTT treatment.

### Functional effects of MTT and lanreotide treatment on cell viability

**Fig 2:**
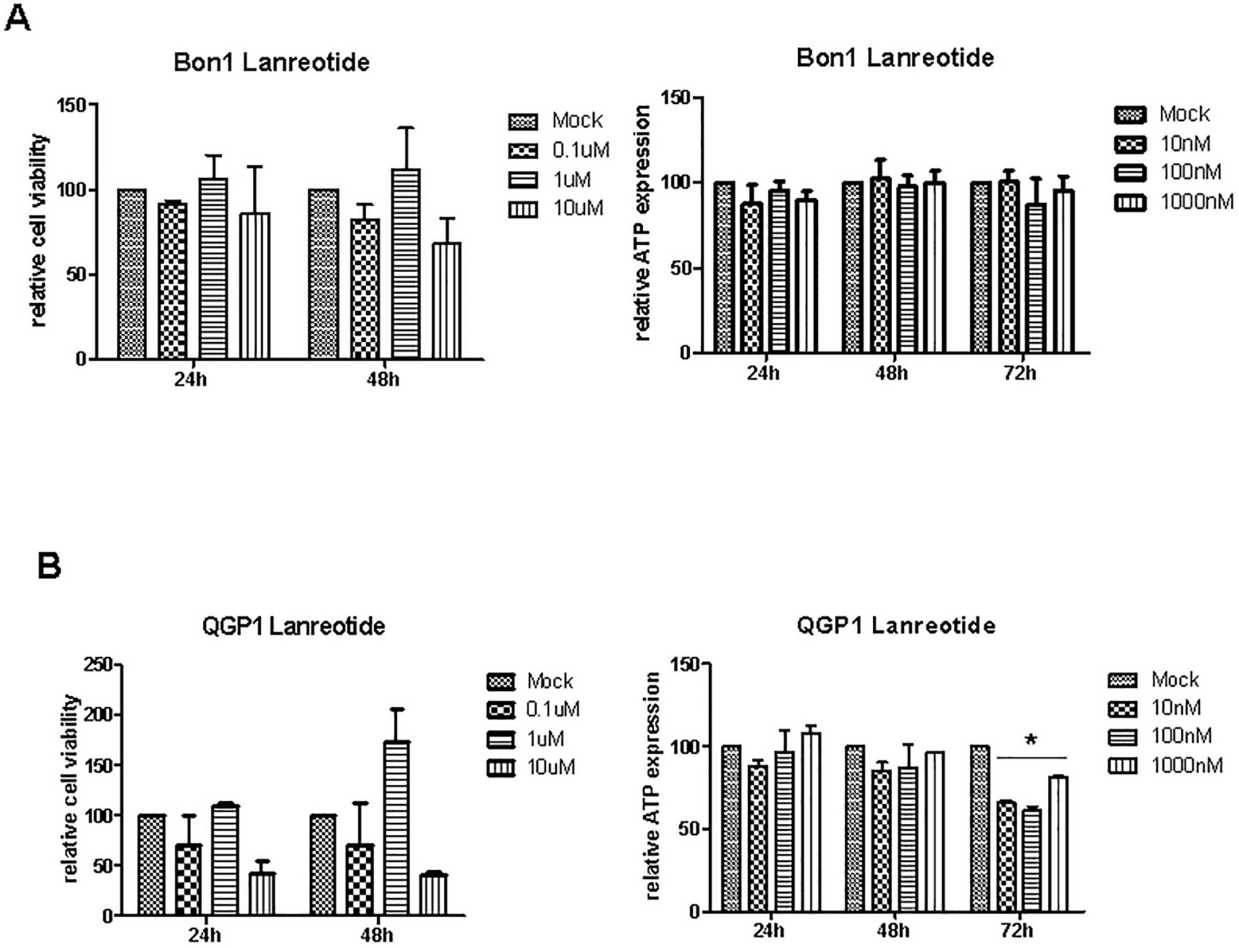
Cell viability studies in Bon1 (A) and QGP1 (B) cells treated with lanreotide (A). Relative cell viability (MTT assay) and ATP expression is presented in at least three independent experiments. * P < 0.05.

Cell toxicity and viability assays were used to measure cell viability in Bon1 and QGP1 cells following different treatment strategies (Fig 2 and S4 Fig).

Lanreotide treatment was performed in different application schedules, including repetitive applications and prolonged incubation time periods for both pancreatic NET cell lines. Viability assays showed no influence of various treatment doses and application schedules on cell viability in Bon1 cells (Fig 2A). In QGP1 cells, only prolonged treatment over 72 hours led to a significantly reduced viability (Fig 2B). Additional cell cycle analysis via flow cytometry did not reveal significant changes in cell cycle progression following lanreotide treatment (S4A Fig) in either cell line. Moreover, no morphological differences could be detected during treatment with lanreotide in both cell lines (S4B and S4C Fig), indicating that lanreotide alone did not affect viability in a relevant manner.

**Fig 3:**
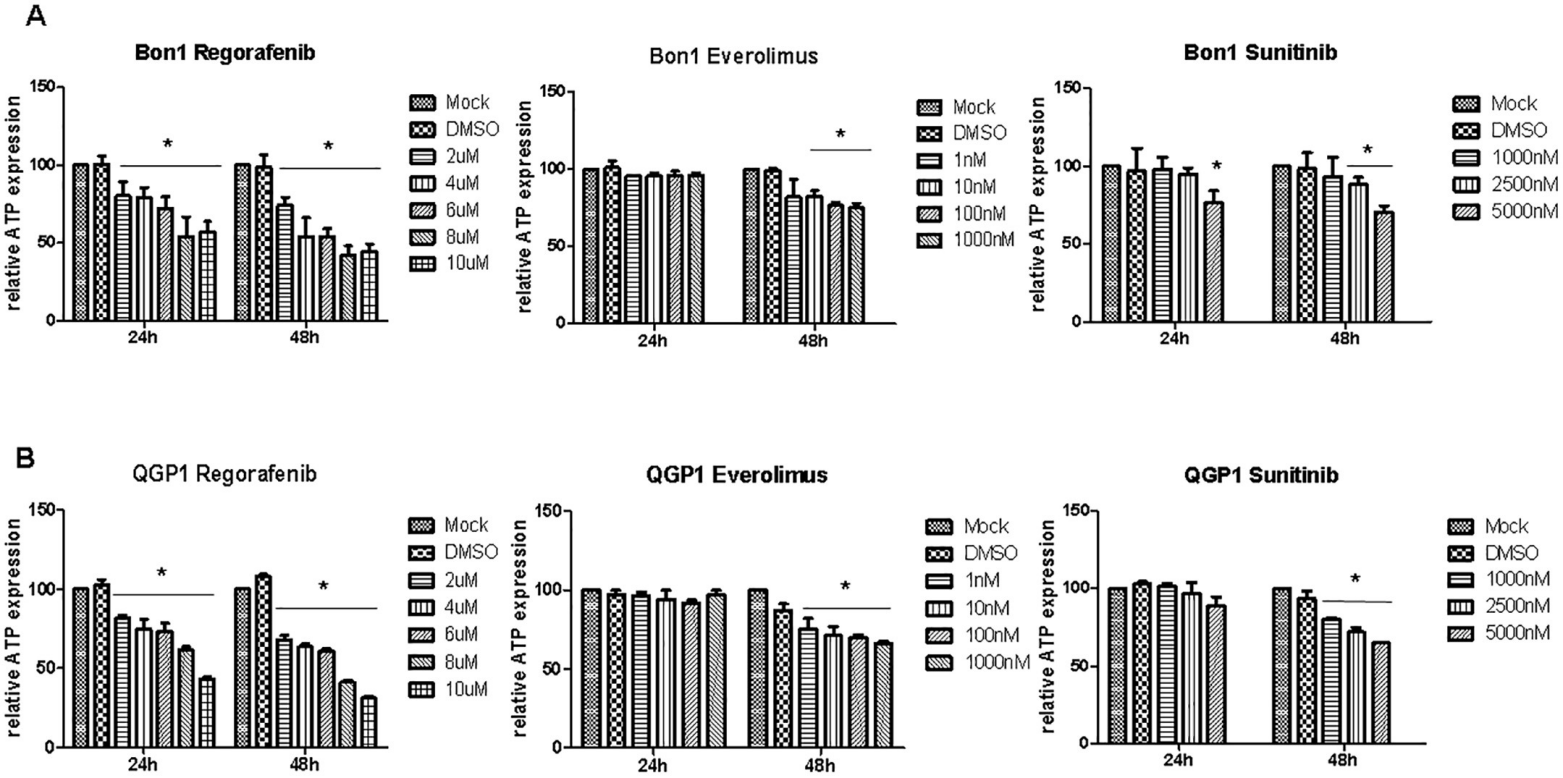
Cell viability studies using ATP assay in Bon1 (A) and QGP1 (B) cells treated with regorafenib, everolimus and sunitinib. Relative cell viability (MTT assay) and ATP expression is presented in at least three independent experiments. * P < 0.05.

In contrast to the SSA-analogue lanreotide, all tested MTT significantly affected cell viability: Regorafenib reduced the cell viability to a maximum of 50% after 48h. Sunitinib and everolimus also diminished the cell viability in both cell lines, however to a lesser extent ranging between 10% and 35% (Fig 3).

### Impact of MTT and lanreotide treatment on intracellular signaling pathways and chromogranin A

**Fig 4:**
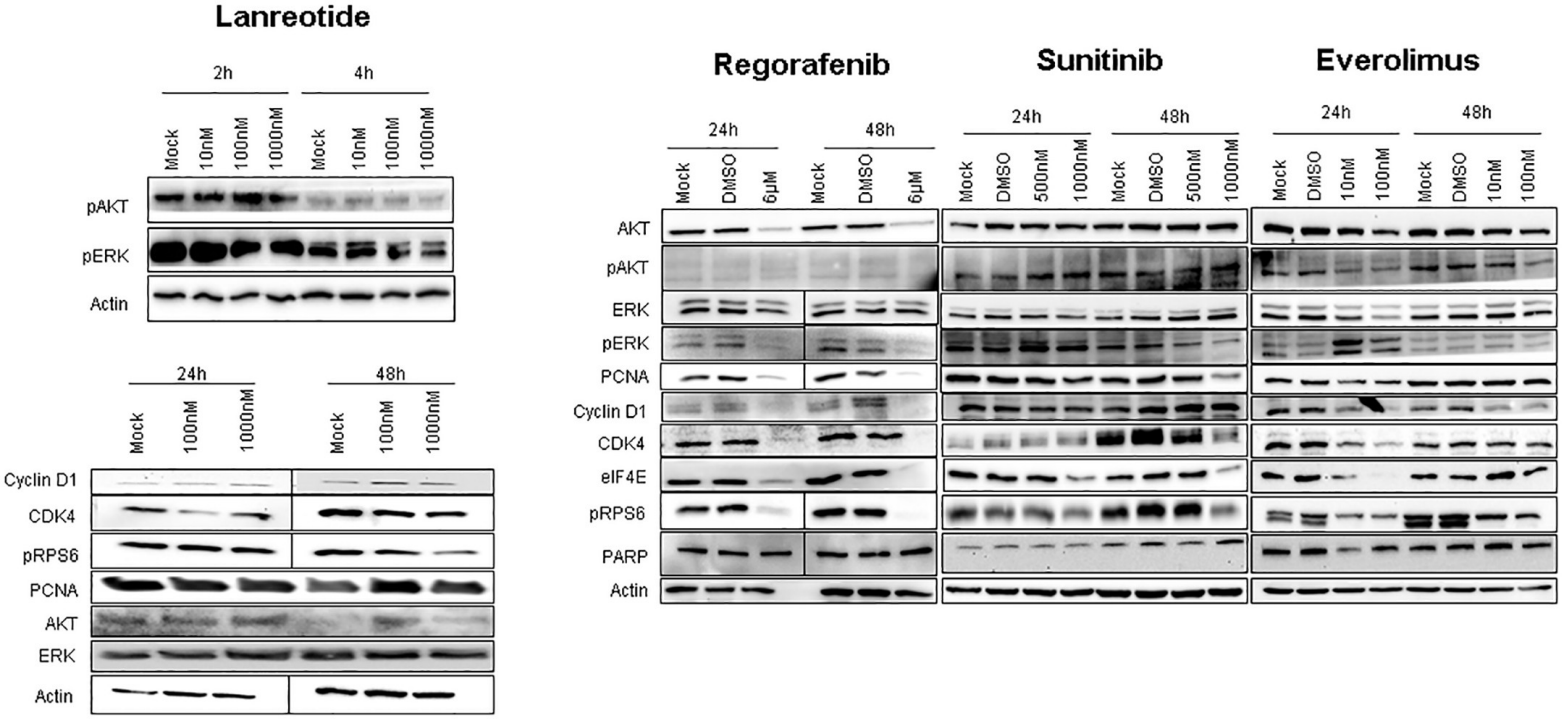
Representative western blots of signaling pathways in Bon1 cells treated with lanreotide, regorafenib, everolimus and sunitinib in different doses. Protein lysates were collected after 24h and 48h for regorafenib, everolimus and sunitinib and after 2h, 4h, 24h and 48h for lanreotide. Data are representative for at least three independent experiments. ß-actin served as internal control.

To assess the impact of MTT and SSA treatment on signaling pathways related to tumor progression, we assessed alterations in ERK, phospho-ERK, PCNA, CDK1 and cyclin D1 for proliferation and cell cycle progression; AKT, phospho-AKT and PARP cleavage for survival and resistance to apoptosis; and phospho-RPS6 and eIF4E for tumor cell growth and protein synthesis, each in Bon1- (Fig 4) and QGP1 cells (S5 Fig) during treatment with lanreotide and MTT. In Bon1 cells, regorafenib induced the most prominent effects on markers of proliferation and cell cycle progression. However, no signs of apoptosis, as assessed by PARP cleavage, could be detected even after 48 hours. As expected, the mTOR inhibitor everolimus markedly decreased phosphorylation of RPS6, a marker of protein synthesis, and exhibited moderate effects on cell cycle progression and proliferation markers (Fig 4). As for regorafenib, no signs of apoptotic cell death were observed. Sunitinib treatment induced a moderate reduction in protein translation markers and delayed effects on proliferation markers after 48 hours (Fig 4). In contrast, the SSA analog lanreotide exerted only a modest effect on AKT and ERK phosphorylation four hours after treatment. Phosphorylation of RPS6 was reduced after 24 hours. All other markers for cell cycle progression or survival were largely unaffected.

Similar to our findings in Bon1 cells, all MTT’s affected PCNA, pRPS6 and pERK in QGP1 cells (S5 Fig). Effects on PARP cleavage and pAKT/AKT could not be detected. Treatment with the SSA analog lanreotide decreased pRPS6 phosphorylation after 48h and 72h, as observed in Bon1 cells (S5 Fig).

**Fig 5:**
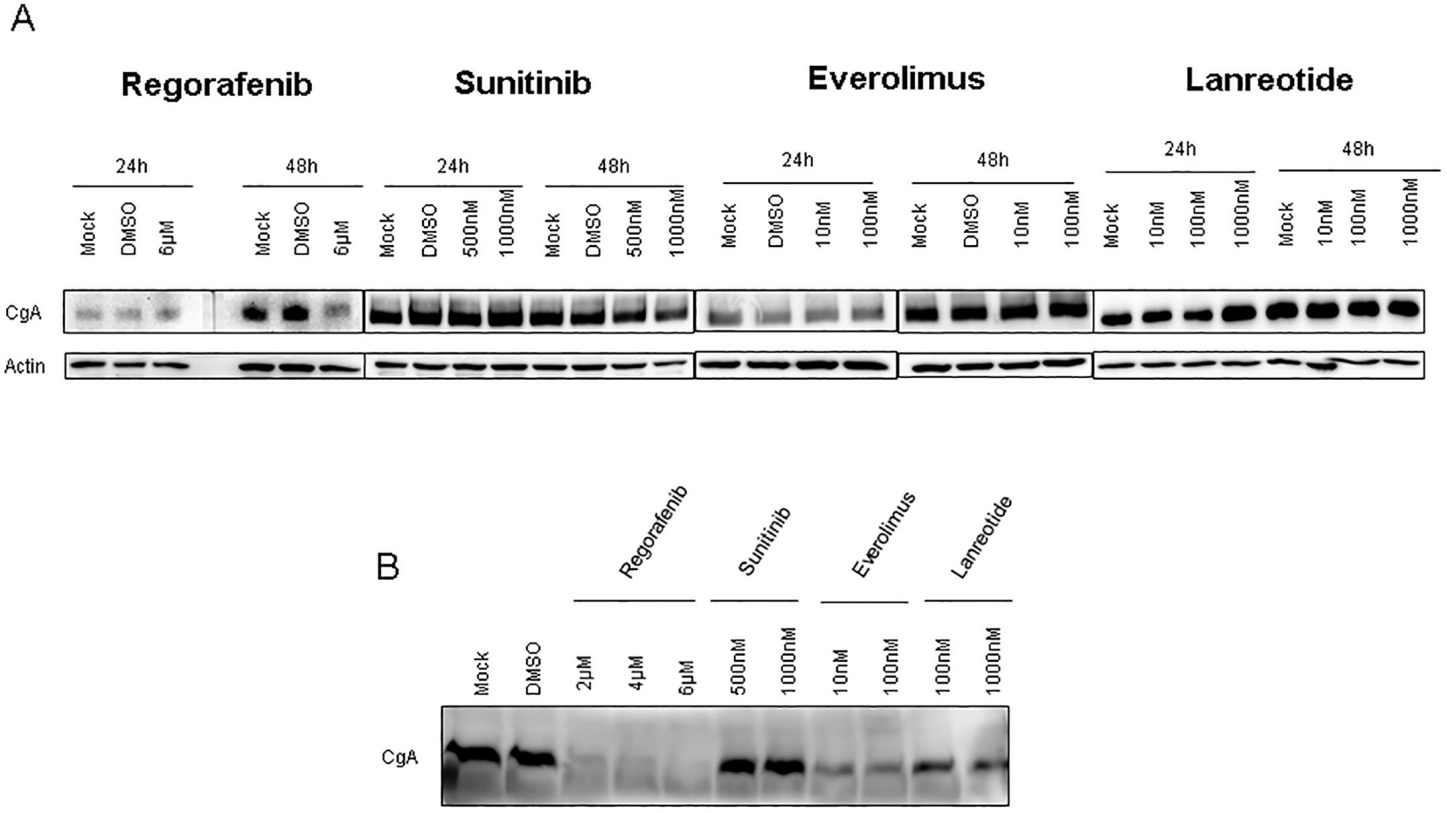
Investigation of protein lysates (A) and supernatant (B) for chromogranin A (CgA) in Bon1 cells treated with the SSA lanreotide and MTT. **(A)** CgA was measured 24h and 48h after treatment with ß-actin as control. **(B)** Supernatant was collected 48h after therapy induction from the same experimental approach shown in **(A)**.

In addition to its anti-proliferative effects, lanreotide is used in clinical routine to treat patients with hormone-related symptoms. Chromogranin A serves as biomarker in patients with GEP-NETs and may also reflect tumor burden in patients with liver metastases [20]. Therefore, we assessed changes in chromogranin A (CgA) levels in both lysates and the supernatants of Bon1 cells, to detect both stored and secreted Chromogranin A. Regorafenib slightly reduced CgA measured in cell lysates after 48 hours. All other MTT’s and lanreotide did not affect the expression of intracellular CgA (Fig 5A). In contrast, secreted CgA was significantly influenced by regorafenib, everolimus and lanreotide (Fig 5B). Even in doses which did not affected cell viability, lanreotide and everolimus treatment led to a marked reduction of CgA in the supernatant, thereby highlighting their anti-secretory capabilities. The decrease of CgA by regorafenib treatment was associated with reduced cell viability, as seen in Fig 3.

### Combined treatment with lanreotide and MTT in different sequences

Since regorafenib and everolimus had the strongest impacts on both cell viability and tumor-related signaling cascades, the combination of lanreotide and both MTT’s was evaluated in further experiments.

**Fig 6:**
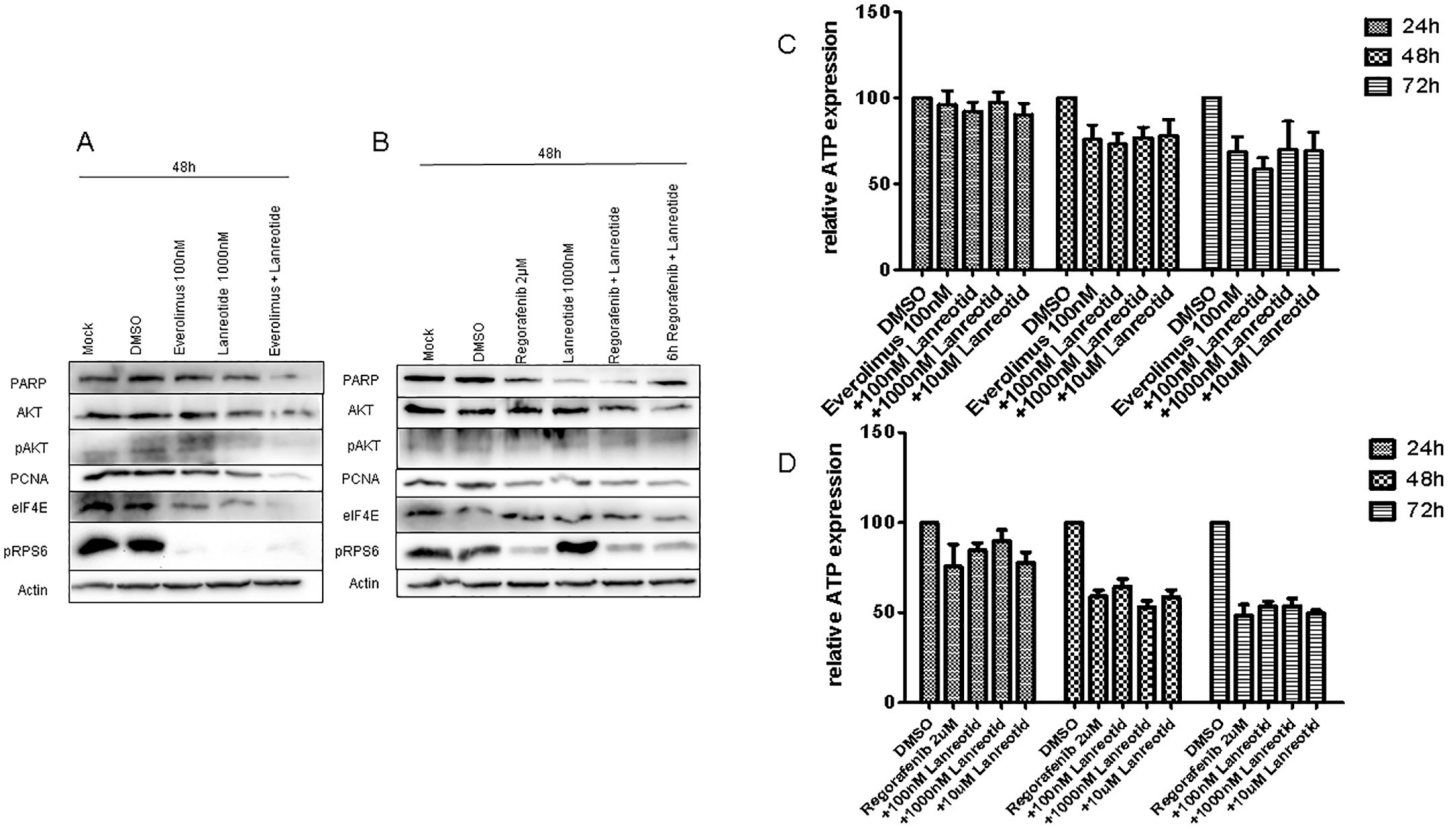
Simultaneous treatment of everolimus plus by lanreotide for 48h (A) and combination treatment of regorafenib plus or followed by lanreotide for 48h in Bon1 cells. Combination treatment of everolimus 100nM (C) or regorafenib 2μM (D) followed by different doses of lanreotide (100nM, 1000nM, 10μM) for 24h to 72h in Bon1 cells. DMSO served as control. Relative ATP expression is presented in %.

In Bon1 cells, the combination of everolimus and lanreotide resulted in a synergistic effect after 48 hours on both PCNA and eIF4E levels compared to monotherapy with either compound (Fig 6A). In contrast, RPS6 phosphorylation was already almost completely abolished by everolimus without further impact of lanreotide (Fig 6A). Similar effects could be detected in QGP cells with synergistic effects being present for PCNA and eIF4E after 48h in the everolimus plus lanreotide group (S6A Fig).

Since our initial results with regorafenib had indicated a transient increase in SSTR2 and -5 expression after 24h incubation (Fig 1), we used simultaneous and also sequential approaches by pretreating Bon1 cells for 6 hours with regorafenib to increase SSTR2 and -5 expression and thereby improve lanreotide efficacy.

Only very moderate synergistic effects could be detected for eIF4E and PCNA after regorafenib pretreatment (Fig 6B). Other proliferative or anti-apoptotic proteins remained unchanged both in the simultaneous and sequential combination treatments. Similar results were detected in QGP1 cells (S6B Fig). Again the combination of regorafenib and lanreotide failed to induce enhanced efficacy. Neither a synchronous nor a metachronous approach demonstrated significant synergisms.

Overall, the combination of lanreotide and MTT yielded only small synergistic effects on intracellular proliferation and growth pathways. This small impact did not translate on synergistic effects on cell viability experiments (Fig 6A and 6B).

## Discussion

It is common clinical practice to combine SSA with anticancer agents including MTT, both for symptom control and for tumor growth control. The rationale for co-administration of SSA and targeted agents in NETs is based on the hypothesis that this drug combination may increase antitumor activity beyond the effects of monotherapy by targeting several proliferative and survival pathway at different levels. So far, however, comprehensive analyses dissecting the crosstalk between SSA and MTT in NET are missing. Our data indicate that some targeted therapies such as regorafenib or everolimus may modulate the SSTR expression pattern. This, however, was not associated with synergistic anti-tumor effects when administered together with the SSA lanreotide *in vitro*.

NET cells express a variety of surface receptors that may induce activating or inhibitory signaling cascades and can be modulated therapeutically. While ligands such as SSA, IFN or chimeric drugs such as dopastatins induce antitumoral effects via binding to inhibitory receptors, activation of receptors for fibroblast growth factor (FGF), insulin-like growth factor-1 (IGF-1), platelet-derived growth factor (PDGF), vascular endothelial growth factor (VEGF), epidermal growth factor (EGF) and transforming growth factors α and β (TGFα/β) induces signal transduction cascades enhancing DNA synthesis, cell proliferation and/or endothelial cell activation [21–23]. These receptor cascades have also been identified as major determinants during tumor progression of NET [24]. The pathways that have been investigated most extensively as therapeutic targets in NET include the somatostatin receptor signaling pathway, the VEGF receptor pathway and the PI3K-Akt-mTOR pathway.

Our preclinical study reveals many limitations, which means that the transfer of the results into clinical practice must not be completely ruled out. The available cell models do not reflect the pathological and molecular biological situation of a well differentiated neuroendocrine G1-G2 tumor. The proliferative capacity of the cell lines used is significantly higher. The drugs used would not be approved in patients with NEN G3 and would only be applicable in an individual concept. Moreover, our in-vitro approach reflects only one compartment of NEN. The tumor stroma including the different known immune and endothelial cells cannot be considered. Thus, there is no possibility to evaluate tumor-cell autonomous SSA and MTT effects. In our study we focused on the investigation of the signaling pathways of PI3K-Akt-mTOR and MAPK. However, it is described in the literature that SSA can trigger indirect tumor suppressive effects via further growth hormones and IGF1 [25].

The extent to which the dose of SSA and its administration scheme have an influence on tumor progression and SSTR regulation cannot be conclusively clarified. Several studies revealed a distinct pattern of receptor desensitization and internalization in response to agonist stimulation [26]. The desensitization may produce a marked shift in the potency of somatostatin stimulation or can lead to a decrease in the maximum inhibition achieved. However, desensitization usually does not lead to a complete absence of responsiveness. The same applies to the internalization of the SSTR. Even if preclinical data show a decrease in SSTR under SSA, no loss of efficacy can be postulated here either. The long PFS times of the CLARINET study in the lanreotide arm are an indication for this [27].

In our preclinical approach the question of desensitization, internalization and heterodimerization of SSTR can only be insufficiently clarified. The discrepancy between tumor cell models with regard to SSTR expression and tumor biology including proliferation capacity is certainly important. With very low effects on tumor cell proliferation and unclear situation regarding drug stability in the cell culture medium, proliferation alone can negate the effects of internalization and heterodimerization.

SSA such as octreotide and lanreotide represent established therapies both for symptom and tumor control which are considered as the first “targeted drug” used in clinical routine. More recently, targeted therapies in NET have focused on the inhibitors of the VEGF and mTOR signaling pathways [14, 15]. The RADIANT-2 trial presented data about the combination of SSA and targeted therapy in a phase III setting [28]. This trial failed to reach statistical significance, however, a clear trend towards improved response rates and prolonged median progression-free survival (mPFS) of combined SSA treatment + VEGF inhibition compared to monotherapy SSA was observed. Post-hoc analysis investigated the influence of previous SSA therapy prior to study entry [29], since many patients had received SSA (~80%) due to carcinoid symptoms. The effect of combination therapy was strongest in SSA naïve patients (25.2 vs. 13.6m), with less efficacy in pretreated patients (14.3 vs. 11.1m).

Interestingly, previous work hypothesized that everolimus positively impacted on the SSA pharmacokinetics [30]. The putative molecular mechanism by which everolimus has been reported to modulate SSA levels is not known. However, it was proposed that inhibition of SSA transporters in the liver and kidney were involved. In contrast to these reports, our *in vitro* data suggest that everolimus leads to downregulation of SSTR5 and exerts its impact on tumor growth irrespective of SSA exposure.

*In vivo* studies to further assess the role of SSA combined with targeted therapies are in part still ongoing, e.g. the SUNLAND study (NCT01731925). However, in the recently published COOPERATE-2 trial pasireotide plus everolimus was investigated in patients with progressive pancreatic NET [31]. According to final results, no benefit was detected in the combination arm regarding PFS (16.8m in the combinations arm vs. 16.6.m in everolimus monotherapy arm). Interestingly, despite a high proportion of G2 tumors in the study, both treatment arms showed longer mPFS rates than previously published in the RADIANT-3 trial [15], which makes interpretation of the results somewhat difficult. In the LUNA trial primary endpoint was the proportion of patients with disease control after 9 months and not mPFS in patients with advanced carcinoids of the lung and thymus [32]. As performed in the COOPERATE-2 trial, similar treatment arms were used: pasireotide LAR 60mg monthly, oral everolimus 10mg daily, and pasireotide LAR plus everolimus. The study endpoint was achieved by 39%, 33.3% and 58.5%, respectively. Based on these encouraging results, further studies need to clarify the anti-proliferative and survival impact of the combination approach.

## Conclusions

In summary, our *in vitro* study data indicate that some targeted therapies are able to modulate the SSTR expression pattern to some extent. This, however, is not associated with synergistic anti-tumor effects when administered simultaneously or sequentially with the SSA lanreotide *in vitro*. Further preclinical and clinical studies are needed to identify potential biomarkers which may select patients for combination therapy approaches with MTT and somatostatin analogues.

## Supporting information

S1 Fig Western Blot analysis (A) and qRT-PCR results (n = 3) of SSTR1-5 in Bon1, QGP1 and Ins-1 cells. For qRT-PCR absolute values are presented.
(TIF)

S2 Fig qRT-PCR triplicates of Bon1 and QGP1 cells treated with lanreotide, regorafenib, everolimus and sunitinib for 24h and 48h. Mock or DMSO control was used. For qRT-PCR absolute values are presented for SSTR1,2 and 5.
(TIF)

S3 Fig qRT-PCR triplicates of Bon1 and QGP1 cells treated with lanreotide, regorafenib, everolimus and sunitinib for 24h and 48h. Mock or DMSO control was used. For qRT-PCR absolute values are presented for SSTR1,2 and 5.
(TIF)

S4 Fig Cell cycle progression using flow cytometry was measured in Bon1 cells after treatment with lanreotide for 48h and 72h. Representative pictures of Bon1 (B) and QGP1 (C) cells during the treatment interval (24h) are shown.
(TIF)

S5 Fig Western blot studies of signaling pathways in QGP1 cells treated with lanreotide, regorafenib, everolimus and sunitinib in different doses. Protein lysates were collected after 24h and 48h for regorafenib, everolimus and sunitinib and after 24h to 72h for lanreotide. Data are representative for at least three independent experiments. ß-actin served as internal control.
(TIF)

S6 Fig Simultaneous treatment of everolimus plus lanreotide for 48h (A) and combination treatment of regorafenib followed by lanreotide for 48h (B) in QGP1 cells. Mock and DMSO served as control.
(TIF)
