# Correction: Interaction between somatostatin analogues and targeted therapies in neuroendocrine tumor cells

**DOI:** 10.1371/journal.pone.0228905

**Published:** 2020-02-04

**Authors:** Sebastian Krug, Jan-Philipp Mordhorst, Fabian Moser, Katharina Theuerkorn, Claudia Ruffert, Maren Egidi, Anja Rinke, Thomas M. Gress, Patrick Michl

Fig 1B shows the presentation of a faulty actin control. The authors have provided a complete, corrected version of Fig 1 here.

**Fig 1:**
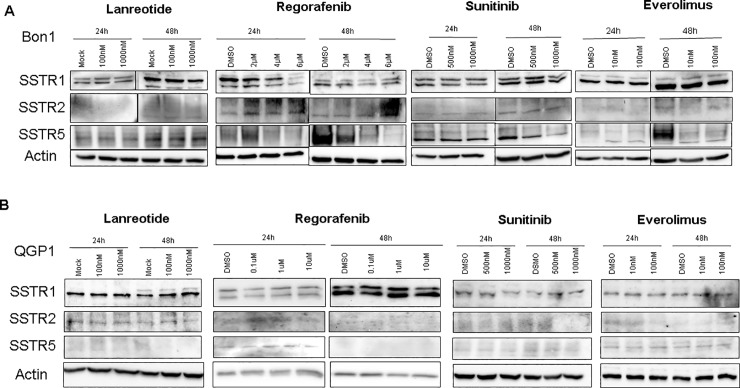
Representative western blots of SSTR1, 2 and 5 in Bon1 and QGP1 cells treated with lanreotide, regorafenib, everolimus and sunitinib in different doses. Protein lysates were collected after 24h and 48h. Data are representative for at least three independent experiments. ß-actin served as internal control.

In the Material and cell lines subsection of the Material and methods section, there is an error in the first sentence. The correct first sentence is: All media contained 10% fetal bovine serum.

In the RNA isolation, cDNA synthesis, and real-time PCR subsection of the Material and methods section, there is an error in the first sentence. The correct first sentence is: RNA was extracted using the RNeasy Mini Kit (Qiagen), and first-strand cDNA was synthesized using primers and Superscript II reverse transcriptase (Invitrogen).

In the Immunoblotting subsection of the Material and methods section, the second sentence is incorrect and should not be present.
