# Supplementary figures and images for: Interaction between somatostatin analogues and targeted therapies in neuroendocrine tumor cells
Source: PLoS One. 2019 Jun 25;14(6):e0218953. doi: 10.1371/journal.pone.0218953 (PMC6592550; doi:10.1371/journal.pone.0218953)

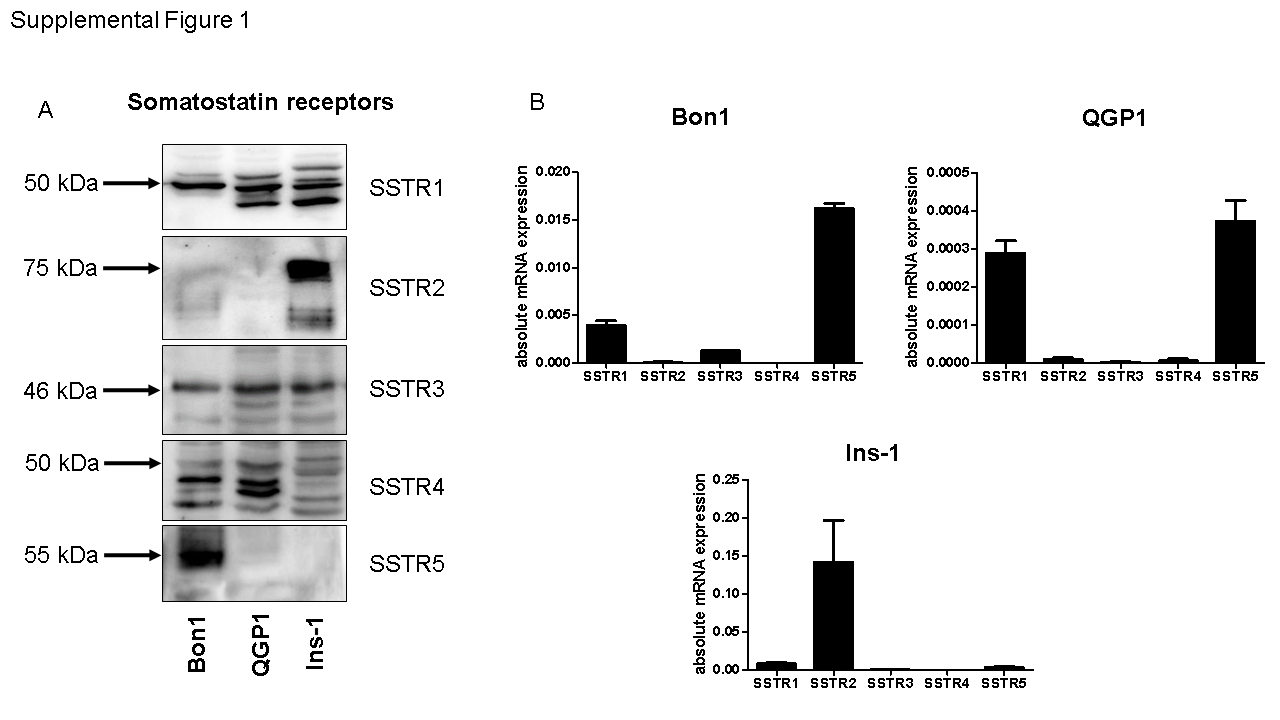

Supplement: S1 Fig — For qRT-PCR absolute values are presented. (TIF) [file pone.0218953.s001.TIF]

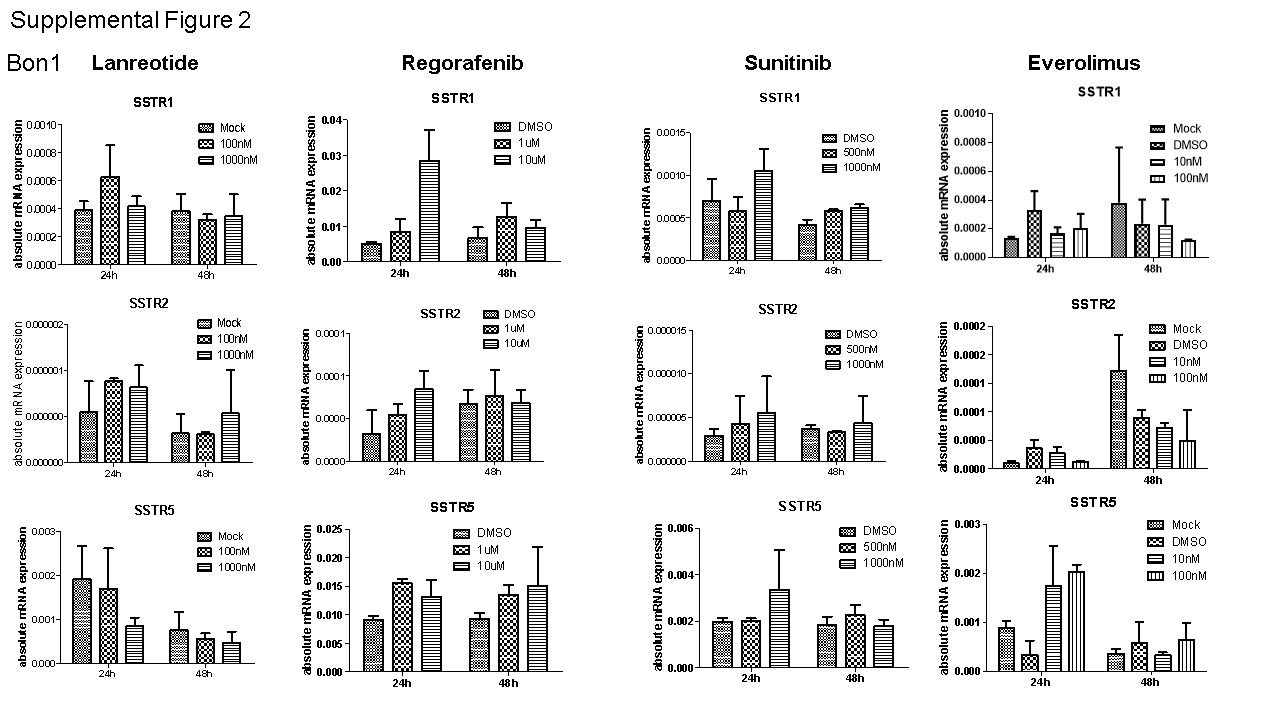

Supplement: S2 Fig — Mock or DMSO control was used. For qRT-PCR absolute values are presented for SSTR1,2 and 5. (TIF) [file pone.0218953.s002.TIF]

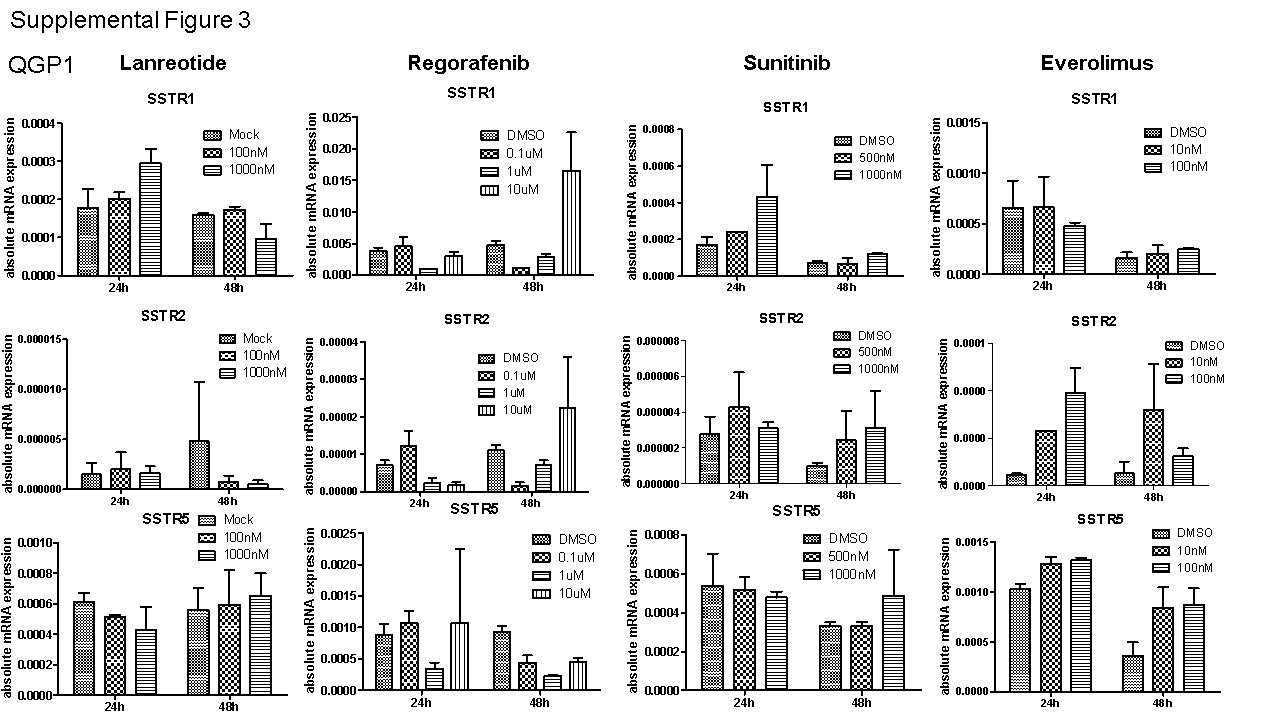

Supplement: S3 Fig — Mock or DMSO control was used. For qRT-PCR absolute values are presented for SSTR1,2 and 5. (TIF) [file pone.0218953.s003.TIF]

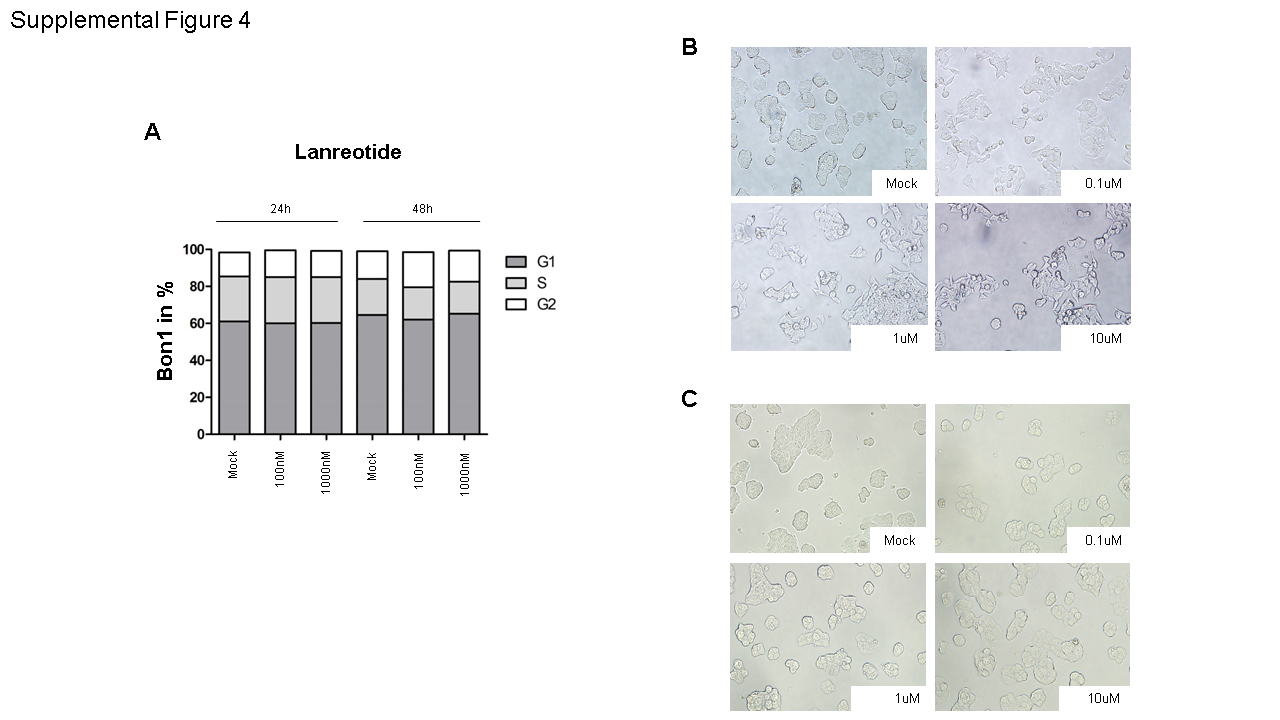

Supplement: S4 Fig — Representative pictures of Bon1 (B) and QGP1 (C) cells during the treatment interval (24h) are shown. (TIF) [file pone.0218953.s004.TIF]

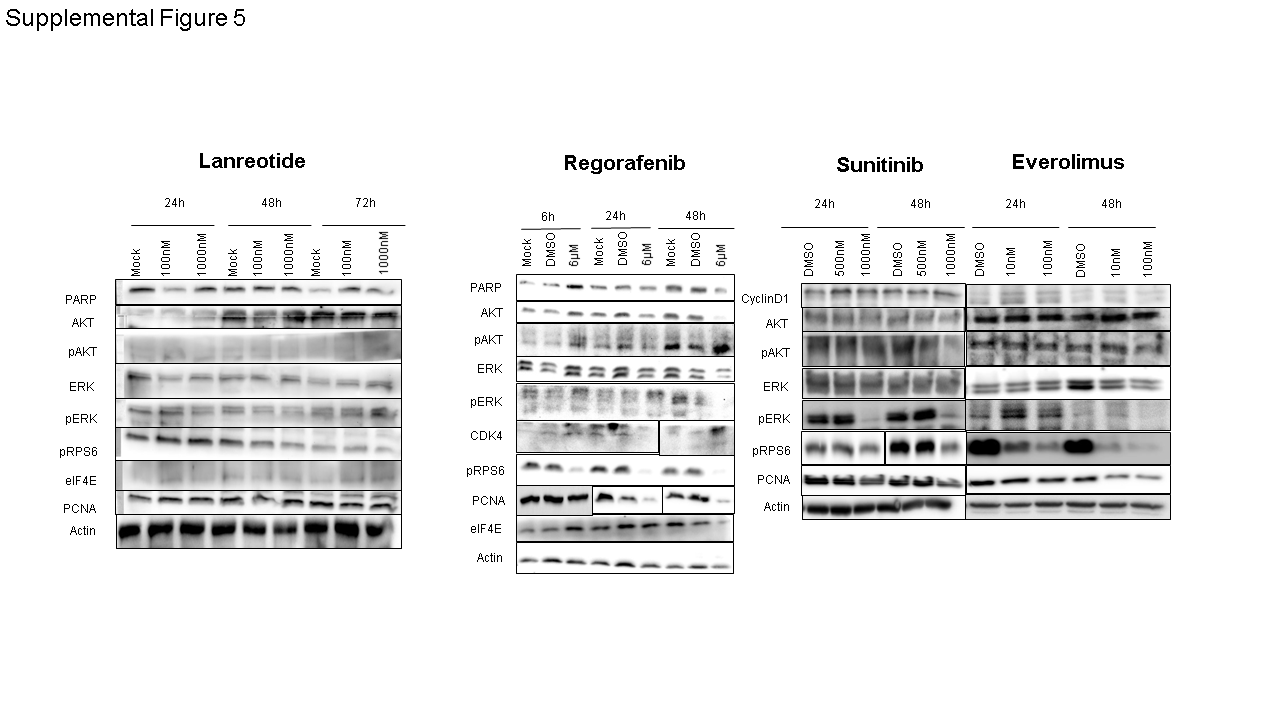

Supplement: S5 Fig — Protein lysates were collected after 24h and 48h for regorafenib, everolimus and sunitinib and after 24h to 72h for lanreotide. Data are representative for at least three independent experiments. ß-actin served as internal control. (TIF) [file pone.0218953.s005.TIF]

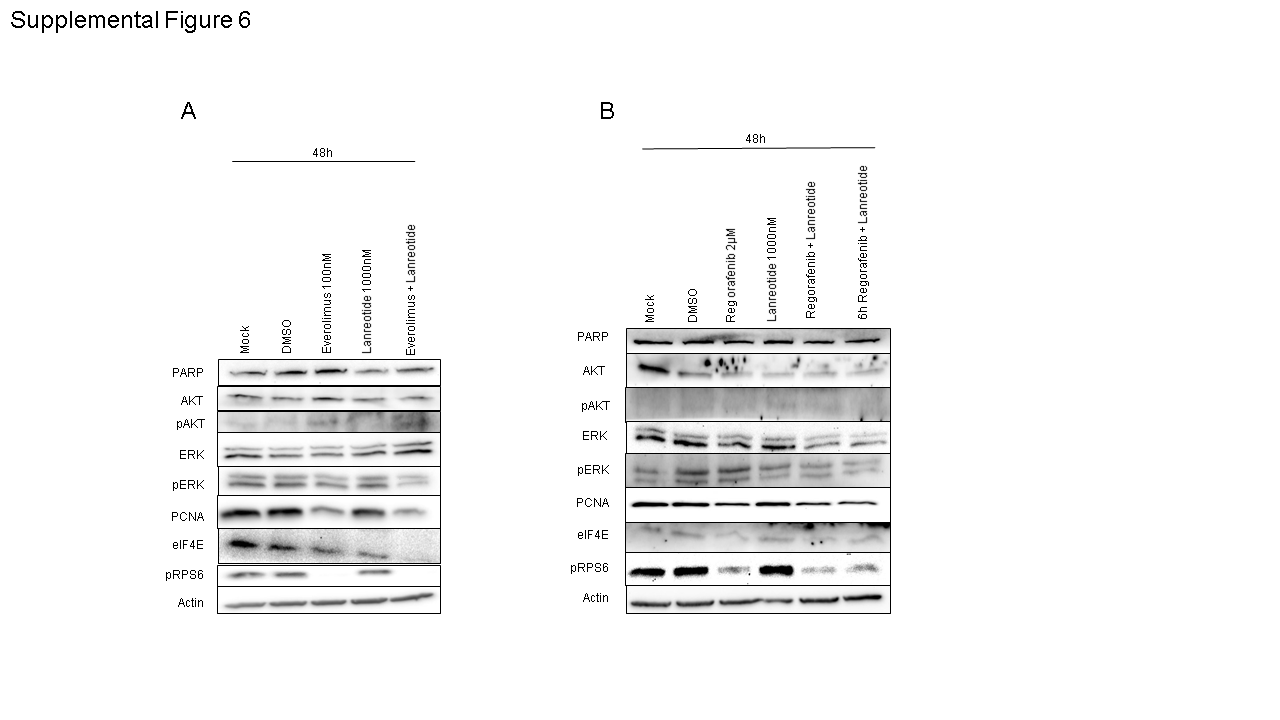

Supplement: S6 Fig — Mock and DMSO served as control. (TIF) [file pone.0218953.s006.TIF]
